# Evaluation of the Relationship between Salivary Lipids, Proteins and Total Antioxidant Capacity with Gingival Health Status in Type-1 Diabetic Children

**DOI:** 10.30476/DENTJODS.2020.84180.1075

**Published:** 2021-06

**Authors:** Fatemeh Tabatabaei, Soleiman Mahjoub, Morteza Alijanpour, Amene Moslemnejad, Samaneh Gharekhani, Forough Yavarzade, Soraya Khafri

**Affiliations:** 1 Dental Student, Student's Research Committee, Babol University of Medical Sciences, Babol, Iran; 2 Cellular and Molecular Biology Research Center, Health Research Institute, Clinical Biochemistry, Babol University of Medical Sciences, Babol, Iran; 3 Non-Communicable Pediatric Diseases Research Center, Health Research Institute, Babol University of Medical Sciences, Babol, Iran; 4 Clinical Biochemistry, Babol University of Medical Sciences, Babol, Iran; 5 Oral Health Research Center, Dept. of Pediatric Dentistry, Faculty of Dentistry, Babol University of Medical Sciences, Babol, Iran; 6 Biostatistics & Epidemiology, Medicine Faculty, Babol University of Medical Sciences, Babol, Iran

**Keywords:** Diabetes mellitus, Saliva, Gingivitis, Children

## Abstract

**Statement of the Problem:**

Alteration in salivary composition and its effect on the oral cavity in diabetic child patients remains equivocal.

**Purpose:**

This study was performed to assess the relationship between salivary factors and gingival status in children with type-1 diabetes mellitus (DM).

**Material and Method:**

In this cross-sectional study, 120 subjects aged 6-16 years (60 well-controlled and poorly-controlled diabetics and 60 healthy individuals) were examined to determine the gingival index (GI) and plaque index (PI). The unstimulated saliva samples were collected to measure the salivary triglyceride, cholesterol, albumin, α-amylase, total protein levels by the laboratory kits. Total antioxidant capacity and the free radicals scavenger index were measured by the Ferric Reducing Ability Of Plasma (FRAP) and 1,1-Diphenyl-2-picryl-hydrazyl (DPPH) assays, respectively. Data were analyzed by parametric and non-parametric, Pearson correlation, and t tests at a 5% error level.

**Results:**

GI of diabetics was significantly higher than that of healthy individuals (1.51± 0.71 and 0.9±0.81, respectively, *p*< 0.001). No significant difference was found between the PI of diabetics compared to healthy volunteers (1.59±0.69, 1.63±0.74, respectively). The levels of salivary triglyceride and cholesterol, albumin and total proteins in healthy subjects were significantly higher than that in people with DM (*p*< 0.001). A significantly more salivary α-amylase activity was found in diabetics compared to non-diabetics (*p*< 0.001). No significant differences were found between diabetic and non-diabetic subjects in terms of DPPH (95.5, 95.9%, respectively) and FRAP (9.77±0.13, 9.78±0.12 (µmol/mL), respectively).

**Conclusion:**

More gingival inflammation and salivary α-amylase activity and lower level of salivary lipids, albumin, and total proteins were found in diabetic patients, but there was no association between the level of lipids, proteins, and the total antioxidant capacity of saliva with periodontal health indicators in patients with DM and healthy individuals.

## Introduction

Diabetes mellitus (DM) is a group of metabolic disorders with serious complications reducing the quality of life [ 1- 2]. It is caused by the absolute or relative insulin deficiency due to decreased secretion of this hormone from the pancreas (type-1 DM) or insensitivity of environmental receptors to this hormone (type-2 DM) [ 3]. The global diabetes prevalence in 2019 is estimated to be 9.3% (463 million people) [ 4]. Type-1 DM usually occurs in childhood, and adolescence includes 5-10% of all diabetes patients [ 3]. 

Periodontal diseases and gingivitis were reported as the sixth most common complication of diabetes, which are usually associated with the severity of disease [ 5- 6]. Some studies showed more prevalence of gingival inflammation in children and adolescents with type-1 DM compared to the healthy population [ 7- 9].

The use of saliva instead of serum has recently been preferred as a diagnostic medium. Saliva offers advantages over blood because it is a cost-effective and non-invasive method that can be collected by persons with modest education [ 10]. One approach to early diagnosis of periodontitis is the salivary biomarkers. Some biomarkers, such as cytokines, were diagnosed and proposed in the literatures [ 11- 13].

Accumulation of reactive oxygen species, oxidative stress, and interactions between advanced glycation end products (AGEs) in the periodontal tissues and their receptor (RAGE) all contribute to increased inflammation in the periodontal tissues in people with DM [ 14]. There is also a lipid metabolism disorder in diabetic patients due to impaired glucose metabolism and changes in insulin secretion and activity. As a result of systemic lipid disorders, high concentrations of lipids have been shown in these patients' blood and saliva. Lipids play the role of nuclei in the dental plaque mineralization and accelerate the activity of the enzyme glucosyltransferase, which is responsible for the carcinogenic activity of oral microorganisms. High cholesterol and triglyceride levels in the plaque, delay the release of lactic acid from it. The presence of lipids in the saliva modulates bacterial hydrophobic surfaces and thus helps to bind them to dental surfaces [ 14- 15]. 

Salivary albumin is regarded as a serum ultrafiltrate to the mouth, and it may diffuse into the mucosal secretions. Hormonal balance, nutrition, and osmotic pressure regulate albumin synthesis. High concentrations of salivary albumin have been detected in a medically compromised condition, such as immunosuppression and DM. Both normal and raised salivary albumin levels have been seen in periodontitis [ 16- 17]. 

In association with α -amylase, some studies have suggested that this salivary enzyme contributes to the microorganisms’ adhesion and the microbial plaque formation; however, other studies have found that α-amylase secretion is associated with a reduced risk of caries, a decrease in oral bacteria, and a reduced risk of periodontal disease [ 18- 20]. DM can affect the composition and flow of saliva. These changes in saliva can be involved in the onset of symptoms and even the severity of oral complications in diabetic patients [ 21]. Many studies have been done about the correlation between salivary composition and periodontal disease in DM, but the results have not been conclusive. Regarding the few studies conducted in this field concerning quality control of the disease, the present study performed to investigate the relationship between periodontal status and salivary protein, lipid, and antioxidant capacity in healthy individuals and patients with well-controlled and uncontrolled type-I DM.

## Materials and Method

### Study population

**Figure 1:**
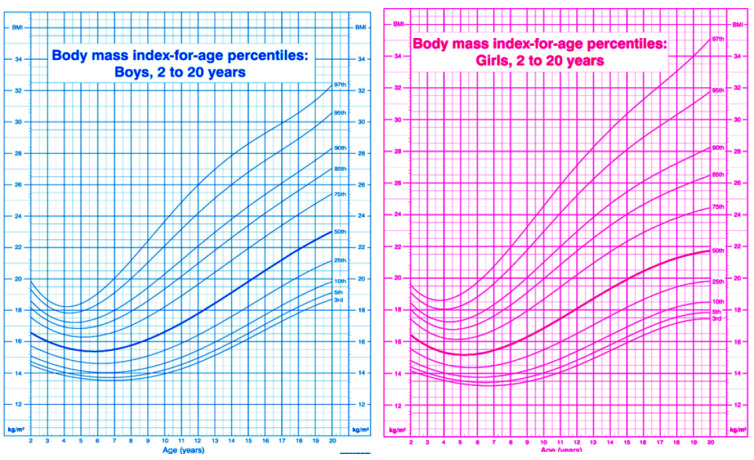
Individual growth chart 3rd, 5th, 10th, 25th, 50th, 75th, 90th, 95th, 97th percentiles, 2 to 20 years: body mass index-for-age [ 22]

This cross-sectional study performed on 6- to 16-year-old diabetic and healthy volunteers with normal body mass index (BMI; percentile 5-85%) (Figure 1) [ 22]. 

Based on previous studies [ 23- 24] and considering an alpha coefficient of 0.05 and a statistical power of 0.8, the sample size was determined to be 120 (case group; n=60 and control group; n=60). The case was divided into two subgroups of well-controlled DM (n=33) and poorly-controlled DM (n=27).

The case group has consisted of patients who were diagnosed with type-1 DM by an endocrinologist at the Amirkola Children Hospital for at least three years. The quality of control of disease was determined based on the level of HbA1c. Patients with HbA1c more than 7.5% were considered into poorly-controlled DM group [ 25]. The patients were selected through a simple sampling method considering the exclusion criteria, such as having other diseases (asthma, cardiovascular disease, epilepsy, and renal deficiency) and reluctance to participate in the study.

The control group included healthy individuals who had not taken any medicine over the last month [ 26]. They were selected from schools of Babol city (north of Iran) using multistage random sampling. According to the different socio-economic situations in various urban districts and their impact on health and nutrition, a multistage sampling method can be generalized to the whole town. However, in the case of patients, because there is only one children's hospital in the city, all the patients can be found in the same place. The subjects in both groups were matched for age and gender.

### Ethical considerations

The study was approved by the Ethical Committee of the Research Council of Babol University of Medical Sciences (MUBABOL.REC.1395.55). The written consent was obtained from all subjects or their parents.

### Experimental procedure

Personal information and medical history of patients were obtained through interviewing participants/ parents and their medical records. In order to minimize the effect of the circadian rhythm, all saliva samples were collected from 10 to 11 AM, and then oral examination was done. Unstimulated saliva samples were collected in disposable sterile tubes and immediately transferred to the laboratory in a container containing dry ice at -4°C. The samples were centrifuged at 1500 g and 15 minutes, Clement 2000, North Sydney, Australia), the supernatant was collected into Eppendorf microtubes and were stored at -80°C until analyzes. 

### Measurement of a lipid profile

Salivary cholesterol and triglyceride levels were assessed based on the colorimetric method using ZiestChem commercial kits (ZiestChem Diagnostics Co.; Iran) according to the manufacturer's protocol [ 27]. 

### Measurement of α-amylase activity

In order to measure the α-amylase activity, 500µl of reagent was poured into the blank and the sample tubes and incubated at 37ºc for 5min. Then 20µl of the sample was added to the sample tube and incubated at 37ºc for 15min. Then immediately after that, the chemical reaction was stopped by adding 500 µl Iodine solution and 1500 µl distilled water. The absorption for the blank and the sample tubes was compared against distilled water at 405nm using UV-visible spectrophotometer, and α-amylase activity was estimated by this equation [ 28]: 

Absorbance of blank-Absorbance of sample Absorbance of blank×1470= α-Amylase activityUL

### Measurement of total protein and albumin concentration

Salivary total protein was measured in the Biuret method using ZiestChem commercial kits (ZiestChem Diagnostics Co., Iran) according to the manufacturer's protocol. Salivary albumin levels were measured based on colorimetric assay using the ZiestChem commercial kit (ZiestChem Diagnostics Co., Iran) also [ 29- 30]. 

### Measurement of FRAP and DPPH indexes

Total antioxidant capacity (TAC) of salvia was measured according to Ferric Reducing Ability of Plasma (FRAP) assay, and the free radicals scavenger index was measured by DPPH assay (1.1-Diphenyl-2-picryl-hydrazyl) [ 31- 32]. Löe and Silness gingival index (GI) and Silness and Löe plaque index (PI) were measured [ 33]. A senior dental student, using a dental mirror and a probe on a chair and in ambient light, did oral examination. 

### Statistical analysis

Data were statistically analyzed by Kruskal-Wallis followed by the Mann-Whitney U Test, One-way analysis of variance (ANOVA), Tukey Scheffe, t test, and Pearson correlation test in SPSS-22. All statistical tests were performed at the significance level of the p value less than 0.05.

## Results

The mean ages of the subjects in case and control groups were respectively 10.02±1.39 and 10.07±0.82 years. Thirty-three diabetic patients (55%) were diagnosed with well-controlled DM (HbA1c less than 7.5%); approximately 4.45±2.43 years elapsed from the diagnosis of DM. The levels of salivary triglyceride and cholesterol, amylase, and GI in people with DM were significantly higher than that in healthy subjects (*p*< 0.001, Table 1).

**Table1:** The mean and standard deviation of the studied variables in the diabetic and healthy subjects

Group	Diabetic subjects	Healthy subjects	*p* Value
Variable			
Triglyceride (mg/dL)[Table-fn t1f1]*	15.82±2.58	7.74±6.98	<0.001
Cholesterol (mg/dL)[Table-fn t1f1]*	11.40±6.49	5.12±3.37	<0.001
Amylase (U/L) [Table-fn t1f1]*	65.40±17.79	42.44±17.56	<0.001
Total protein (g/dL)[Table-fn t1f1]*	91.53±10.82	106.56±11.74	<0.001
Albumin (g/dL)[Table-fn t1f1]*	2.48±0.97	3.10±1.09	<0.001
DPPH (%)[Table-fn t1f1]*	95.5	95.9	0.77
FRAP (µmol/mL)[Table-fn t1f1]*	9.77±0.13	9.78±0.12	0.083
Plaque index[Table-fn t1f2]**	1.63±0.74	1.59±0.69	0.84
Gingival index[Table-fn t1f2]**	1.51±0.74	0.91±0.81	<0.001

* : Based on t-test

** : Based on Mann-Whitney test

Albumin and total proteins in healthy subjects were significantly higher than that in people with DM (*p*< 0.001, Table 1). There was not a significant difference in the amount of salivary antioxidant capacity and PI between healthy and diabetics individuals (Table1). 

In terms of gender, the level of salivary lipids and total proteins in both males (*p*= 0.00) and females of non-diabetic subjects were significantly higher than those of diabetic subjects (*p*< 0.001). Salivary α-amylase activity in both males and females of the case group was significantly higher than that of the control group (*p*< 0.001). Salivary albumin in diabetic men was significantly lower than that of healthy ones (*p*<0.001).

Nevertheless, no significant difference was found between the salivary albumin level of diabetic and non-diabetic females (*p*= 0.11).

Table 2 illustrates the mean and standard deviation or median of study variables in the well-controlled DM and poorly-controlled DM and non-diabetic groups (Table 2). No significant correlation was found between all salivary parameters and gingival status (Table 3).

**Table2:** The mean and standard deviation of the study variables in the well-controlled and poorly- controlled diabetic and healthy subjects

Groups	Poorly-controlled Diabetes	Well-controlled Diabetes	Healthy subjects	*p* Value
Variables				
Triglyceride (mg/dL) [Table-fn t2f1]*	16.42± 3.65^A^	14.23± 8.81^A^	7.74 ±6.98^B^	<0.001
Cholesterol (mg/dL)[Table-fn t2f1]*	11.79±4.19^A^	11.01± 2.59^A^	5.12 ±3.37^B^	<0.001
Amylase(U/L)[Table-fn t2f1]*	63.88 ±18.21^A^	66.64 ±17.94^A^	42.44±17.55^B^	<0.001
Total protein(g/dL)[Table-fn t2f1]*	89.24 ± 10.27^A^	93.39 ±11.05^A^	106.56±11.74^B^	<0.001
Albumin(g/dL)[Table-fn t2f1]*	2.49 ±1.11^A^	2.47± 0.84^A^	3.10±1.09^B^	0.006
DPPH (%)[Table-fn t2f1]*	95.2	95.8	95.9	0.952
FRAP(µmol/mL)[Table-fn t2f1]*	9.76 ±0.11	9.77 ±0.14	9.78±0.12	0.355
Plaque index [Table-fn t2f2]**	1.84± 0.73	1.45± 0.69	1.59±0.69	0.56
Gingival index [Table-fn t2f2]**	1.70 ±0.62	1.26 ±0.93	0.91±0.81	0.133

* : Based on ANOVA and Tukey Scheffe

** : Based on Kruskal Wallis and Mann-Whitney test

A and B: The same capital letters indicate the non-significant difference between every two groups

**Table3:** The [Table-fn t3f1]*correlation of salivary biomarkers with GI and PI in the diabetic and healthy subjects

Group	Variable		Cholesterol	Triglyceride	Total protein	Albumin	Amylase	DPPH	FRAP
Healthy subjects	GI	P	0.592	0.582	0.274	0.869	0.501	0.618	0.960
		R	-0.071	0.072	-0.143	0.022	-0.089	0.0066	-0.007
	PI	P	0.147	0.846	0.313	0.823	0.773	0.628	0.946
		R	-0.189	0.026	-0.133	-0.030	0.033	0.069	0.009
Diabetic subjects	GI	P	0.864	0.152	0.390	0.675	0.243	0.286	0.889
		R	0.023	-0.187	-0.113	0.055	-0.153	0.140	0.018
	PI	P	0.134	0.748	0.772	0.549	0.213	0.185	0.375
		R	0.196	-0.042	-0.038	-0.079	-0.163	0.174	-0.117

* Based on Pearson Correlation Test

## Discussion

This study aimed to evaluate the level of salivary lipids and proteins and TAC of patients with type-1 DM and their correlation with gingival health status compared to healthy children. In the present study, a higher GI was found in diabetic patients compared to non-diabetics. However, there was no significant difference in the PI of study groups. So, gingival inflammation in these patients seems not to be related to oral hygiene.

Similar to this result, Alves *et al*. [ 34] indicated a significant increase in the GI of diabetic patients compared to healthy individuals despite a non-significant difference in plaque index. Machado *et al*. [ 35] found no significant difference between GI of diabetic and non-diabetic subjects, despite a higher PI in patients with DM. The published data on the exclusive influence of microbial plaque on gingival inflammation in patients suffering from type-1 DM are controversial [ 36]. Pathogenesis of DM in gingivitis and periodontitis can be attributed to factors such as small vessels involvement, changes in gingival fluid composition and elevation of inflammatory mediators [ 37], changes in collagen metabolism, decreased defense responses, the increased presence of periodontal pathogenic microorganisms and oxidative stress [ 38] and genetic predisposition to non-enzymatic glycosylation [ 36]. A positive correlation was shown between periodontitis and GI and gingival bleeding/ dental biofilm by Daković and Pavlović [ 36]. They suggested these items as the prognostic indicator of potential periodontitis.

In the present study, the GI of poorly-controlled diabetic children was significantly higher than that of healthy children. Available literature data illustrated a correlation between the incidence and severity of gingival inflammation and poor metabolic control of DM [ 36]. So, control of DM seems to be critical to the prevention of gingival and periodontal disease. 

Sadeghi *et al*. [ 39] showed a higher level of GI in the 13-18 year old diabetic patients, but they found no relationship between the HbA1c level and periodontal indices.

Salivary cholesterol and triglyceride of diabetic patients were estimated lower than that of non-diabetic patients. In contrast, Priya *et al*. [ 4] found a higher level of salivary lipids (cholesterol and triglyceride) in patients with type-1 DM, which might be due to demographic and racial differences. 

In the present study, a higher level of salivary α-amylase was found in DM patients compared to healthy subjects. However, a higher level of salivary total proteins and albumin were observed in healthy individuals. Lakshmi *et al*. [ 41] showed a higher level of salivary total proteins and α-amylase in patients with DM. Panchbhai *et al*. [ 42] studied on salivary total proteins, and α-amylase of well-controlled and poorly-controlled DM patients compared with healthy individuals and showed a significantly lower level of salivary α-amylase in patients with well-controlled DM compared to healthy subjects. However, no significant differences were found between other variables and groups. In the present study, no significant difference was found between well-controlled and poorly-controlled groups about the salivary proteins levels.

The TAC measured by FRAP and radical scavenger index determined by the DPPH assay were not significantly different among study groups. In contrast, Basir *et al*. [ 43] used the TAC kit for measuring the level of salivary antioxidants and reported that patients with type1 DM had less antioxidant defense compared to healthy children. A different method for measurement of salivary antioxidants can be a reason for different results. Astaneie *et al*. [ 44] reported no significant difference between the level of reactive thiobarbituric acid as a lipid peroxidation marker in diabetics and the control group. In a study conducted by Gümüş *et al*. [ 45], the mean salivary reduced-glutathione concentration in type-1 diabetic patients was estimated lower than that of healthy subjects, but there was no significant difference in the concentration of other antioxidants among different groups. Rai *et al*. [ 46] estimated the phosphomolydic acid in saliva using spectrophotometry and showed that salivary antioxidant level was lower in diabetic patients than that of healthy individuals. 

In the present study, it was found no significant correlation between GI and PI and TAC. Aral *et al*. [ 47] reported that the oxidative stress index in diabetic patients was higher than that of the control group. However, it decreased after initiating treatment for DM, and instability in oxidative conditions with DM may be a significant contributor to periodontal disease. 

In the study conducted by Reznick *et al*. [ 48], there was a strong correlation between the severity of DM and the increase of salivary and serum antioxidants such as peroxidase and superoxide dismutase. Overall, measuring the antioxidant agents by different methods can be a reason for diversity in a result of various studies. No correlation was found between PI, salivary lipids, proteins, and TAC with gingival health status. So, the authors suggest that in addition to further studies on these variables, the other risk factors for gingival problems in diabetic patients involve features of inflammation, immune function, neutrophil activity, and cytokine biology to be considered.

## Conclusion

A higher GI was found in diabetic patients compared to healthy children, which was not related to microbial plaque accumulation. Salivary lipids and protein levels despite α-amylase in DM patients were lower than that of healthy subjects, but no difference was found between salivary lipids or protein levels in well-controlled and poorly-controlled patients. TAC of saliva was not significantly different between groups. No correlation was found between salivary lipids, proteins, and TAC with gingival health status.
